# Author Correction: Repulsions instruct synaptic partner matching in an olfactory circuit

**DOI:** 10.1038/s41586-025-10089-9

**Published:** 2026-01-04

**Authors:** Zhuoran Li, Cheng Lyu, Chuanyun Xu, Ying Hu, David J. Luginbuhl, Asaf B. Caspi-Lebovic, Jessica M. Priest, Engin Özkan, Liqun Luo

**Affiliations:** 1 Department of Biology and Howard Hughes Medical Institute, Stanford University, Stanford, CA USA; 2 Department of Biochemistry and Molecular Biology, The Neuroscience Institute and Institute for Biophysical Dynamics, The University of Chicago, Chicago, IL USA

**Keywords:** Axon and dendritic guidance, Olfactory system

Correction to: *Nature* 10.1038/s41586-025-09768-4 Published online 19 November 2025

Since the version of the article initially published, in the “Generation of cDNA constructs and transgenic flies” section of the Methods and in Supplementary Table 1, the transgenic fly used for experiments in Fig. 3g and i for Toll2 overexpression has been corrected: the transgenic fly was generated using the plasmid pJFRC19-13XLexAop2-IVS-Toll2-Flag and carries the LexAop-Toll2-Flag transgene on the third chromosome (VK5 site). This correction has been made to the HTML and PDF versions of the article.

