# Supplementary material for: Repulsions instruct synaptic partner matching in an olfactory circuit
Source: Nature. 2025 Nov 19;649(8097):667–76. doi: 10.1038/s41586-025-09768-4 (PMC12804089; doi:10.1038/s41586-025-09768-4)
Supplement: Supplementary file 1 — This file contains Supplementary Data 1: Raw data for Extended Data Fig. 8d,e; Supplementary Table 1: Summary of genotypes used in each experiment, arranged according to figure panels; and Supplementary References. [file 41586_2025_9768_MOESM1_ESM.pdf]

nature portfolio

https://doi.org/10.1038/s41586-025-09768-4

Supplementary information

Repulsions instruct synaptic partner matching in an olfactory circuit

In the format provided by the
authors and unedited

Supplementary Information Guide

Repulsions instruct synaptic partner matching in an olfactory circuit

Zhuoran Li[1,3], Cheng Lyu[1,3], Chuanyun Xu[1], Ying Hu[1], David J. Luginbuhl[1], Asaf B. Caspi-Lebovic[2], Jessica M. Priest[2], Engin Özkan[2], Liqun Luo[1]*

[1] Department of Biology and Howard Hughes Medical Institute, Stanford University, Stanford, CA 94305, USA
[2] Department of Biochemistry and Molecular Biology, The Neuroscience Institute and Institute for Biophysical Dynamics, The University of Chicago, Chicago, IL 60637, USA
[3] These authors contributed equally
* Corresponding author. Email: lluo@stanford.edu

Supplementary Data 1. Raw data for Extended Data Fig. 8d, e.

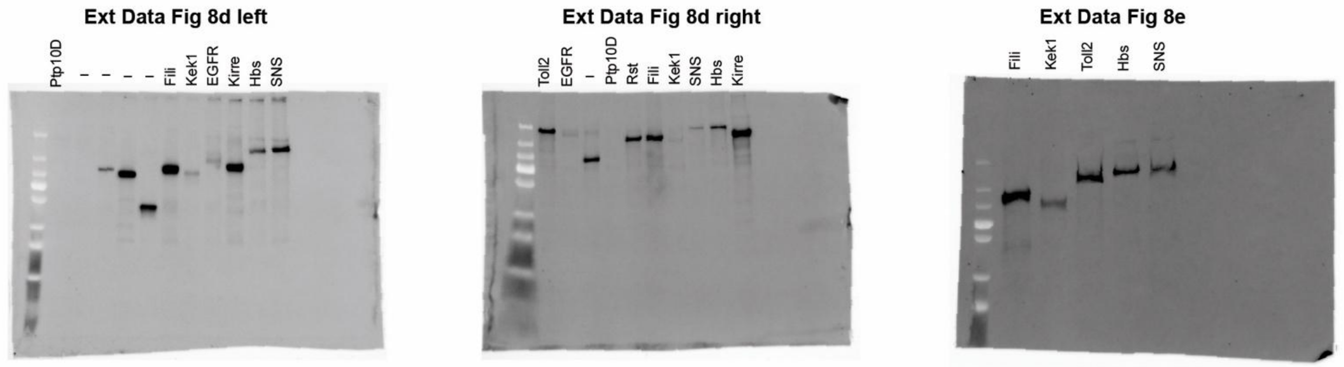

Supplementary Table 1

Figure and panel	Brief description	Fly genotype		
		x chromosome	2nd chromosome	3rd chromosome
**Figure 1**				
c	Ptp10D in ORNs	*Ptp10D>HA>Myc (this study) / eyFLP*	*UAS>stop>mCD8-GFP (BDSC 30125) / +*	
	Ptp10D in PNs	*Ptp10D>HA>Myc*	*UAS-FLP (BDSC 4539) / +*	*VT033006-GAL4*[1] */ +*
d	Toll2 in ORNs	*eyFLP*[2]	*Toll2>HA>Myc (this study) / UAS>stop>mCD8-GFP*	
	Toll2 in PNs		*Toll2>HA>Myc / UAS-FLP*	*VT033006-GAL4 / +*
f	Fili in ORNs	*eyFLP*	*Fili>HA>Myc (this study) / UAS>stop>mCD8-GFP*	
	Kek1 in PNs		*kek1>HA>Myc (this study) / UAS-FLP*	*VT033006-GAL4 / +*
	Kek1 in ORNs	*eyFLP*	*kek1>HA>Myc / UAS>stop>mCD8-GFP*	
g	Hbs in PNs		*hbs>HA>Myc (this study) / UAS-FLP*	*VT033006-GAL4 / +*
	Sns in PNs		*sns>HA>Myc (this study) / UAS-FLP*	*VT033006-GAL4 / +*
	Kirre in ORNs	*kirre>HA>Myc (this study) / eyFLP*	*UAS>stop>mCD8-GFP / +*	
	Kirre in PNs	*kirre>HA>Myc*	*UAS-FLP / +*	*VT033006-GAL4 / +*
**Figure 2**				
b	VA1d-ORN Control[3]	*UAS-Dcr2* (BDSC 24646), *UAS-mCD8-GFP (BDSC 24648)*	*R20D10-QF2*[3]*, QUAS-mtdTomato-HA (BDSC 30004) / +*	*R78H05-p65.AD (BDSC 601815), R31F09-GAL4.DBD (BDSC 68759) / +*
c	*$Ptp10D^{-}$/Y*	*Ptp10D[1]*[4] (BDSC 5810) / *Y*	*Mz19-Gal4 (BDSC 34497), UAS-mCD8-GFP*[5]*, Or88a-mtdt*[6] */ +*	*Or47b-CD2 (BDSC 9915) / +*
d	VA1d-ORN > *Ptp10D* RNAi	*UAS-Dcr2, UAS-mCD8-GFP*	*R20D10-QF2, QUAS-mtdTomato-HA (BDSC 30004) / +*	*R78H05-p65.AD, R31F09-GAL4.DBD / UAS-Ptp10D-RNAi (BDSC 39001)*
e	Single VA1d-ORN > *Ptp10D* RNAi	*hsFLP*[7]*, UAS-mCD8-GFP*		*R78H05-FRT100-stop-FRT100-p65.AD (this study), R31F09-GAL4.DBD / UAS-Ptp10D-RNAi (BDSC 39001)*
f	*$Toll2^{+/-}$*		*18w[Delta7-35]* [8] *(BDSC 4372) / +*	*Or88a-mtdTomato*[6]*, Or47b-rCD2 / +*
g	VA1v-PN > *Toll2* RNAi	*UAS-Dcr2, UAS-mCD8-GFP*	*Or88a-mtdTomato, Or47b-CD2 (BDSC 9916) / +*	*Dop1R2-VP16.AD*[9] *(gift from C. Desplan lab), VT033006-GAL4.DBD (BDSC 73333) / UAS-Toll2-RNAi (BDSC 30498)*
h	VA1d-PN Control[3]	*UAS-Dcr2, UAS-mCD8-GFP*	*R73F07-p65.AD (BDSC 70805) / +*	*R26E12-GAL4.DBD (BDSC 70157) / +*
i	VA1d-PN > *Ptp10D* RNAi	*UAS-Dcr2, UAS-mCD8-GFP*	*R73F07-p65.AD / +*	*R26E12-GAL4.DBD / UAS-Ptp10D-RNAi (BDSC 39001)*
j	VA1v-ORN > *Toll2* RNAi	*UAS-Dcr2, UAS-mCD8-GFP*	*VT043692-p65.AD (BDSC 72503), R20D10-QF2, QUAS-mtdTomato-HA/ +*	*CG11317-GAL4.DBD*[9] *(gift from C. Desplan lab) /*

				UAS-Toll2-RNAi (BDSC 30498)
**Figure 3**				
a	Control	*UAS-Dcr2, UAS-mCD8-GFP*	*R20D10-QF2, QUAS-mtdTomato-HA / +*	*R78H05-p65.AD, R31F09-GAL4.DBD / +*
b	VA1d-ORN > Toll2 OE	*UAS-Dcr2, UAS-mCD8-GFP*	*R20D10-QF2, QUAS-mtdTomato-HA / P{GSV1}18w^{EP-709} (BDSC 43442)*	*R78H05-p65.AD, R31F09-GAL4.DBD / +*
c	*Ptp10D$^{–}$/Y*	*Ptp10D[1] / Y*	*R20D10-QF2, QUAS-mtdTomato-HA / +*	*R78H05-p65.AD, R31F09-GAL4.DBD / +*
d	*Ptp10D$^{–}$/Y*, VA1d-ORN > Toll2 OE	*Ptp10D[1] / Y*	*R20D10-QF2, QUAS-mtdTomato-HA / P{GSV1}18w^{EP-709}*	*R78H05-p65.AD, R31F09-GAL4.DBD / +*
f	Control	*UAS-Dcr2*	*Mz19-AD^{G4HACK}* [3], *lexAop-rCD2::RFP-p10.UAS-mCD8::GFP-p10 (BDSC 67093) / +*	*R26E12-Gal4.DBD, R31F09-LexA.DBD*[3], *R78H05-p65.AD / +*
g	VA1d-ORN > Toll2 OE	*UAS-Dcr2*	*Mz19-AD^{G4HACK}, lexAop-rCD2::RFP-p10.UAS-mCD8::GFP-p10 / +*	*R26E12-Gal4.DBD, R31F09-LexA.DBD, R78H05-p65.AD / LexAop-Toll2-Flag (this study)*
h	VA1d-PN > *Ptp10D* RNAi	*UAS-Dcr2*	*Mz19-AD^{G4HACK}, lexAop-rCD2::RFP-p10.UAS-mCD8::GFP-p10 / +*	*R26E12-Gal4.DBD, R31F09-LexA.DBD, R78H05-p65.AD / UAS-Ptp10D-RNAi (BDSC 39001)*
i	VA1d-ORN > Toll2 OE, VA1d-PN > *Ptp10D* RNAi	*UAS-Dcr2*	*Mz19-AD^{G4HACK}, lexAop-rCD2::RFP-p10.UAS-mCD8::GFP-p10 / +*	*R26E12-Gal4.DBD, R31F09-LexA.DBD, R78H05-p65.AD / UAS-Ptp10D-RNAi (BDSC 39001), LexAop-Toll2-Flag*
**Figure 4**				
b	VA1v-ORN control	*UAS-Dcr2, UAS-mCD8-GFP*	*VT043692-p65.AD / +*	*CG11317-GAL4.DBD / +*
c	*kek1$^{–/–}$*		*kek1 mutant*[10] *(NIG-fly M2L-1096) / kek1 mutant*	*Or47b-GAL4 (BDSC 9984), UAS-myr-GFP-p10 (gift from G. Rubin lab) / +*
d	VA1v-ORN > *kek1* RNAi	*UAS-Dcr2, UAS-mCD8-GFP*	*VT043692-p65.AD / UAS-kek1-RNAi (VDRC 101166)*	*CG11317-GAL4.DBD / +*
f	VA1d-ORN control	*UAS-Dcr2, UAS-mCD8-GFP*	*R20D10-QF2, QUAS-mtdTomato-HA / +*	*R78H05-p65.AD, R31F09-GAL4.DBD / +*
g	VA1d-ORN > Kek1 OE	*UAS-Dcr2, UAS-mCD8-GFP*	*R20D10-QF2, QUAS-mtdTomato-HA / P{GSV1}kek1^{EP-840} (BDSC 43665)*	*R78H05-p65.AD, R31F09-GAL4.DBD / +*
h	Single VA1d-ORN > Kek1 OE	*hsFLP, UAS-mCD8-GFP*	*P{GSV1}kek1^{EP-840} / +*	*R78H05-FRT100-stop-FRT100-p65.AD, R31F09-GAL4.DBD / +*
i	*Fili$^{–/–}$*	*UAS-Dcr2, UAS-mCD8-GFP, QUAS-mtdTomato-HA*[11]	*Fili ex^18*[12] */ FRTG13, Fili ex^18*	*R78H05-p65.AD, R31F09-GAL4.DBD / +*
j	*Fili$^{+/–}$*, VA1d-ORN > Kek1 OE	*UAS-Dcr2, UAS-mCD8-GFP, QUAS-mtdTomato-HA*	*Fili ex^18 / P{GSV1}kek1^{EP-840}*	*R78H05-p65.AD, R31F09-GAL4.DBD / +*
k	*Fili$^{–/–}$*, VA1d-ORN > Kek1 OE	*UAS-Dcr2, UAS-mCD8-GFP, QUAS-mtdTomato-HA*	*Fili ex^18 / FRTG13, Fili ex^18, P{GSV1}kek1^{EP-840}*	*R78H05-p65.AD, R31F09-GAL4.DBD / +*
**Figure 5**				

b	VA1v-ORN control	*UAS-Dcr2, UAS-mCD8-GFP*	*VT043692-p65.AD / +*	*CG11317-GAL4.DBD / +*
c	VA1v-ORN > *kirre* RNAi	*UAS-Dcr2, UAS-mCD8-GFP*	*VT043692-p65.AD / UAS-kirre-RNAi (VDRC 109585)*	*CG11317-GAL4.DBD / +*
d	VA1d/DA1-PN > *hbs* RNAi	*UAS-Dcr2, UAS-mCD8-GFP*	*Mz19-GAL4, UAS-mCD8-GFP, Or88a-mtdTomato / UAS-hbs-RNAi (VDRC 40898)*	*Or47b-CD2 / +*
e	VA1d/DA1-PN > *sns* RNAi	*UAS-Dcr2, UAS-mCD8-GFP*	*Mz19-GAL4, UAS-mCD8-GFP, Or88a-mtdTomato / UAS-sns-RNAi (VDRC 109442)*	*Or47b-CD2 / +*
g	VA1d-ORN control	*UAS-Dcr2, UAS-mCD8-GFP*	*R20D10-QF2, QUAS-mtdTomato-HA / +*	*R78H05-p65.AD, R31F09-GAL4.DBD / +*
h	VA1d-ORN > Kirre OE	*UAS-Dcr2, UAS-mCD8-GFP*	*R20D10-QF2, QUAS-mtdTomato-HA / +*	*R78H05-p65.AD, R31F09-GAL4.DBD / (this study)*
i	Single VA1d-ORN > Kirre OE	*hsFLP, UAS-mCD8-GFP*		*R78H05-FRT100-stop-FRT100-p65.AD, R31F09-GAL4.DBD / UAS-kirre-HA(this study)*
j	VA1d-ORN > Kirre OE, $sns^{-/-}$	*UAS-myr-GFP-p10 (gift from G. Rubin lab)*	*SNS[XB3] / SNS[XB3]*	*R78H05-p65.AD, R31F09-GAL4.DBD / UAS-kirre-HA*
k	VA1d-ORN > Kirre OE, $hbs^{+/-}$	*UAS-myr-GFP-p10*	*hbs[2593]*[14] */ +*	*R78H05-p65.AD, R31F09-GAL4.DBD / UAS-kirre-HA*
l	VA1d-ORN > Kirre OE, $sns^{-/-}$, $hbs^{+/-}$	*UAS-myr-GFP-p10*	*SNS[XB3] / SNS[XB3], hbs[2593]*[14] *(gift from M. Baylies)*	*R78H05-p65.AD, R31F09-GAL4.DBD / UAS-kirre-HA*
m	VA1d-ORN > Kirre OE, *kirre* $^{-}$/Y	$Df(1)duf_{sps-1}$ */ Y*	*UAS-myr-GFP-p10 (gift from G. Rubin lab)*	*R78H05-p65.AD, R31F09-GAL4.DBD / UAS-kirre-HA*
**Extended Data Figure 1**				
a	VA1d-ORN control		*Mz19-QF*[16]*, QUAS-mCD8-GFP*[11]*, Or88a-mtdTomato, Or47b-rCD2 /+*	
b	Pan-ORN > *Ptp10D* RNAi	*Pebbled-GAL4*[17]*, UAS-Dcr2*	*Mz19-QF, QUAS-mCD8-GFP, Or88a-mtdTomato, Or47b-rCD2 / +*	*UAS-Ptp10D-RNAi (BDSC 39001) / +*
c	Pan-PN > *Toll2* RNAi	*UAS-Dcr2*	*Mz19-QF, QUAS-mCD8-GFP, Or88a-mtdTomato, Or47b-rCD2 / +*	*VT033006-GAL4 / UAS-Toll2-RNAi (BDSC 30498)*
d	VA1v-ORN control		*Mz19-QF, QUAS-mCD8-GFP, Or88a-mtdTomato, Or47b-rCD2 /+*	
e	Pan-ORN > *kek1* RNAi	*Pebbled-GAL4, UAS-Dcr2*	*Mz19-QF, QUAS-mCD8-GFP, Or88a-mtdTomato, Or47b-rCD2 / UAS-kek1-RNAi (VDRC 101166)*	
f	Pan-ORN > *kirre* RNAi	*Pebbled-GAL4, UAS-Dcr2*	*Mz19-QF, QUAS-mCD8-GFP, Or88a-mtdTomato, Or47b-rCD2 / UAS-kirre-RNAi (VDRC 109585)*	
g	Pan-neuronal > *hbs* RNAi	*C155-GAL4*[18]*, UAS-Dcr2*	*Mz19-QF, QUAS-mCD8-GFP, Or88a-mtdTomato, Or47b-rCD2 / UAS-hbs-RNAi (VDRC 40898)*	
h	Pan-neuronal > *sns* RNAi	*C155-GAL4, UAS-Dcr2*	*Mz19-QF, QUAS-mCD8-GFP, Or88a-mtdTomato, Or47b-rCD2 / UAS-sns-RNAi (VDRC 109442)*	

**Extended Data Figure 2**	same as Fig. 1 with additional groups listed below			
c	Fili in PNs		*Fili>HA>Myc / UAS-FLP*	*VT033006-GAL4 / +*
	Hbs in ORNs	*eyFLP*	*hbs>HA>Myc / UAS>stop>mCD8-GFP*	
	Sns in ORNs	*eyFLP*	*sns>HA>Myc / UAS>stop>mCD8-GFP*	
**Extended Data Figure 3**				
a	VA1d-ORN > *Ptp10D* RNAi	*UAS-Dcr2, UAS-mCD8-GFP, QUAS-mtdTomato-HA*	*R20D10-QF2 / +*	*R78H05-p65.AD, R31F09-GAL4.DBD / UAS-Ptp10D-RNAi (BDSC 39001)*
b	VA1d-ORN > *Ptp10D* RNAi	*UAS-Dcr2*	*VT003280-LexA (BDSC 94678), lexAop-rCD2::RFP-p10.UAS-mCD8::GFP-p10 / +*	*R78H05-p65.AD, R31F09-GAL4.DBD / UAS-Ptp10D-RNAi (BDSC 39001)*
c	VA1d/DA1-PN > *Ptp10D* RNAi	*UAS-Dcr2, UAS-mCD8-GFP*	*Mz19-GAL4, UAS-mCD8-GFP, Or88a-mtdTomato / +*	*Or47b-CD2 / UAS-Ptp10D-RNAi (BDSC 39001)*
d	pan-ORN > *Toll2* RNAi	*Pebbled-GAL4, UAS-Dcr2*	*Mz19-QF, QUAS-mCD8-GFP, Or88a-mtdTomato, Or47b-rCD2 / +*	*UAS-Toll2-RNAi (BDSC 30498) / +*
e	VA1v-ORN > *Toll2* RNAi	*UAS-Dcr2, UAS-mCD8-GFP*	*VT043692-p65.AD, R20D10-QF2, QUAS-mtdTomato-HA/ +*	*CG11317-GAL4.DBD / UAS-Toll2-RNAi (BDSC 30498)*
f	Control	*UAS-Dcr2, UAS-mCD8-GFP*	*R82E01-QF2*[3]*, QUAS-mtdTomato-HA / +*	*R78H05-p65.AD, R31F09-GAL4.DBD / +*
	VA1d-ORN > *Ten-m* RNAi + Kek1 OE	*UAS-Dcr2, UAS-mCD8-GFP*	*R82E01-QF2, QUAS-mtdTomato-HA / UAS-Ten-m-RNAi (VDRC v330540), P{GSV1}kek1*$^{EP-840}$	*R78H05-p65.AD, R31F09-GAL4.DBD / +*
	VA1d-ORN > *Ten-m* RNAi + Kek1 OE + *Ptp10D* RNAi	*UAS-Dcr2, UAS-mCD8-GFP*	*R82E01-QF2, QUAS-mtdTomato-HA / UAS-Ten-m-RNAi (VDRC v330540), P{GSV1}kek1*$^{EP-840}$	*R78H05-p65.AD, R31F09-GAL4.DBD / UAS-Ptp10D-RNAi (BDSC 39001)*
**Extended Data Figure 4**				
b-d	VA1d-ORN sparse labeling control	*hsFLP, UAS-mCD8-GFP*		*R78H05-FRT100-stop-FRT100-p65.AD, R31F09-GAL4.DBD / +*
h	VA1v-ORN driver		*UAS-myr-GFP-p10 / VT043692-p65.AD*	*UAS-myr-GFP-p10 / CG11317-GAL4.DBD*
i	VA1v-PN driver		*UAS-myr-GFP-p10 / +*	*UAS-myr-GFP-p10 / Dop1R2-VP16.AD, VT033006-GAL4.DBD*
**Extended Data Figure 5**				
a	VA1d-ORN > Toll2 OE	*UAS-Dcr2, UAS-mCD8-GFP*	*R20D10-QF2, QUAS-mtdTomato-HA / +*	*R78H05-p65.AD, R31F09-GAL4.DBD / UAS-Toll2-Flag (this study)*
b	VA1d-ORN > Toll2 OE		*R20D10-QF2, QUAS-mtdTomato-HA / P{GSV1}18w*$^{EP-709}$*, Or43a-mCD8-GFP (BDSC 52625)*	*R78H05-p65.AD, R31F09-GAL4.DBD / +*
c	VA1v-ORN > Ptp10D OE	*UAS-Dcr2, UAS-mCD8-GFP*	*VT043692-p65.AD / +*	*CG11317-GAL4.DBD / UAS-Ptp10D-HA (this study)*

**Extended Data Figure 6**				
a	*kirre⁻/Y*	*Df(1)duf sps-1*[13] *(gift from M. Ruiz) / Y*		*Or47b-GAL4, UAS-myr-GFP-p10 / +*
b	*hbs+/–*		*FRT42D, hbs[66]*[14] *(BDSC 27618) / +*	*Or47b-GAL4, UAS-myr-GFP-p10 / +*
c	*sns–/–*		*FRT42D, sns[XB3]*[15] */ FRT42D, sns[XB3]*	*Or47b-GAL4, UAS-myr-GFP-p10 / +*
d	VA1d-ORN > Kek1 OE	*UAS-Dcr2, UAS-mCD8-GFP, QUAS-mtdTomato-HA*	*P{GSV1}kek1EP-840 / +*	*R78H05-p65.AD, R31F09-GAL4.DBD / +*
	VA1d-ORN > Kek1 OE, *sns+/–*, *hbs+/–*	*UAS-Dcr2, UAS-mCD8-GFP, QUAS-mtdTomato-HA*	*P{GSV1}kek1EP-840 / SNS[XB3], hbs[2593]*	*R78H05-p65.AD, R31F09-GAL4.DBD / +*
**Extended Data Figure 7**				
a	VA1d-ORN > *Ptp10D* RNAi	*UAS-Dcr2, UAS-mCD8-GFP / UAS-Brp-Short-mStraw*[19]*, UAS-mCD8-GFP*	*R20D10-QF2, QUAS-mtdTomato-HA / +*	*R78H05-p65.AD, R31F09-GAL4.DBD / UAS-Ptp10D-RNAi (BDSC 39001)*
b	VA1d-ORN > Kek1 OE	*UAS-Dcr2, UAS-mCD8-GFP / UAS-Brp-Short-mStraw*[19]*, UAS-mCD8-GFP*	*R20D10-QF2, QUAS-mtdTomato-HA / P{GSV1}kek1EP-840*	*R78H05-p65.AD, R31F09-GAL4.DBD / +*
c	VA1d-ORN > Kirre OE	*UAS-Dcr2, UAS-mCD8-GFP / UAS-Brp-Short-mStraw*[19]*, UAS-mCD8-GFP*	*R20D10-QF2, QUAS-mtdTomato-HA / +*	*R78H05-p65.AD, R31F09-GAL4.DBD / UAS-kirre-HA*
**Extended Data Figure 9**	genotypes labeled on top of images			
**Extended Data Figure 10**				
a	VMSC control[12]		*R86C10-LexA, LexAop-mtdTomato, Or98-mCD8-GFP, Or92a-rCD2*[12] */ +*	*VT033006-GAL4 / +*
b	pan-PN > *kek1* RNAi		*R86C10-LexA, LexAop-mtdTomato, Or98-mCD8-GFP, Or92a-rCD2 / UAS-kek1-RNAi (VDRC 101166)*	
d	DA1-ORN control[20]	*UAS-Dcr2, UAS-mCD8-GFP*	*VT028327-p65.AD (BDSC 73064), Mz19-QF2*[20]*, QUAS-mtdTomato-HA / +*	*R22E04-GAL4.DBD (BDSC 69199) / +*
	DA1-ORN > Kirre OE	*UAS-Dcr2, UAS-mCD8-GFP*	*VT028327-p65.AD, Mz19-QF2, QUAS-mtdTomato-HA / +*	*R22E04-GAL4.DBD*[20] */ P{EP}kirre[G876] (BDSC 26609)*
e	DA4l/VA1d-ORN control[3]	*UAS-Dcr2, UAS-mCD8-GFP*	*VT023830-p65.AD (BDSC 72467) / +*	*R31F09-GAL4.DBD / +*
	DA4l/VA1d-ORN > Kirre OE	*UAS-Dcr2, UAS-mCD8-GFP*	*VT023830-p65.AD / +*	*R31F09-GAL4.DBD / UAS-kirre-HA*
**Extended Data Figure 11**				
a	homozygous conditional tagging flies (this study) for each gene			

b	Ptp10D and Toll2 co-staining	*Ptp10D>HA>Myc*	*Toll2>V5>Flag (this study) / +*	
	Kek1 and Fili co-staining		*Kek1>HA>Myc / Fili>V5>Flag (this study)*	
	Kirre and Hbs co-staining	*Kirre>V5>Flag (this study)*	*Hbs>HA>Myc / +*	

References:

1 Tirian, L. & Dickson, B. J. The VT GAL4, LexA, and split-GAL4 driver line collections for targeted expression in the Drosophila nervous system. *BioRxiv*, 198648 (2017).

2 Newsome, T. P., Asling, B. & Dickson, B. J. Analysis of Drosophila photoreceptor axon guidance in eye-specific mosaics. *Development* **127**, 851-860 (2000).

3 Lyu, C. *et al.* Dimensionality reduction simplifies synaptic partner matching in an olfactory circuit. *Science* **388**, 538-544 (2025).

4 Sun, Q., Bahri, S., Schmid, A., Chia, W. & Zinn, K. Receptor tyrosine phosphatases regulate axon guidance across the midline of the Drosophila embryo. *Development* **127**, 801-812 (2000).

5 Lee, T. & Luo, L. Mosaic analysis with a repressible cell marker for studies of gene function in neuronal morphogenesis. *Neuron* **22**, 451-461 (1999).

6 Ward, A., Hong, W., Favaloro, V. & Luo, L. Toll receptors instruct axon and dendrite targeting and participate in synaptic partner matching in a Drosophila olfactory circuit. *Neuron* **85**, 1013-1028 (2015).

7 Golic, K. G. & Lindquist, S. The FLP recombinase of yeast catalyzes site-specific recombination in the Drosophila genome. *Cell* **59**, 499-509 (1989).

8 Eldon, E. *et al.* The Drosophila 18 wheeler is required for morphogenesis and has striking similarities to Toll. *Development* **120**, 885-899 (1994).

9 Chen, Y. D. *et al.* Using single-cell RNA sequencing to generate predictive cell-type-specific split-GAL4 reagents throughout development. *Proc Natl Acad Sci USA* **120**, e2307451120 (2023).

10 Kondo, S. & Ueda, R. Highly improved gene targeting by germline-specific Cas9 expression in Drosophila. *Genetics* **195**, 715-721 (2013).

11 Potter, C. J., Tasic, B., Russler, E. V., Liang, L. & Luo, L. The Q system: a repressible binary system for transgene expression, lineage tracing, and mosaic analysis. *Cell* **141**, 536-548 (2010).

12 Xie, Q. *et al.* Transsynaptic Fish-lips signaling prevents misconnections between nonsynaptic partner olfactory neurons. *Proc Natl Acad Sci USA* **116**, 16068-16073 (2019).

13 Weavers, H. *et al.* The insect nephrocyte is a podocyte-like cell with a filtration slit diaphragm. *Nature* **457**, 322-326 (2009).

14 Artero, R. D., Castanon, I. & Baylies, M. K. The immunoglobulin-like protein Hibris functions as a dose-dependent regulator of myoblast fusion and is differentially controlled by Ras and Notch signaling. *Development* **128**, 4251-4264 (2001).

15 Bour, B. A., Chakravarti, M., West, J. M. & Abmayr, S. M. Drosophila SNS, a member of the immunoglobulin superfamily that is essential for myoblast fusion. *Genes Dev* **14**, 1498-1511 (2000).

16 Hong, W., Mosca, T. J. & Luo, L. Teneurins instruct synaptic partner matching in an olfactory map. *Nature* **484**, 201-207 (2012).

17 Sweeney, L. B. *et al.* Temporal target restriction of olfactory receptor neurons by Semaphorin-1a/PlexinA-mediated axon-axon interactions. *Neuron* **53**, 185-200 (2007).

18 Kurusu, M. *et al.* A screen of cell-surface molecules identifies leucine-rich repeat proteins as key mediators of synaptic target selection. *Neuron* **59**, 972-985 (2008).

19 Fouquet, W. *et al.* Maturation of active zone assembly by Drosophila Bruchpilot. *J Cell Biol* **186**, 129-145 (2009).

20 Xu, C. *et al*. Molecular and cellular mechanisms of teneurin signaling in synaptic partner matching. *Cell* **187**, 5081-5101.e5019 (2024).
