# Supplementary material for: Repulsions instruct synaptic partner matching in an olfactory circuit
Source: Nature. 2025 Nov 19;649(8097):667–76. doi: 10.1038/s41586-025-09768-4 (PMC12804089; doi:10.1038/s41586-025-09768-4)
Supplement: Supplementary file 2 — Reporting Summary [file 41586_2025_9768_MOESM2_ESM.pdf]

nature portfolio

Corresponding author(s): Liqun Luo

Last updated by author(s): Sep 16, 2025

Reporting Summary

Nature Portfolio wishes to improve the reproducibility of the work that we publish. This form provides structure for consistency and transparency in reporting. For further information on Nature Portfolio policies, see our Editorial Policies and the Editorial Policy Checklist.

Statistics

For all statistical analyses, confirm that the following items are present in the figure legend, table legend, main text, or Methods section.

n/a	Confirmed	
☐	☒	The exact sample size (*n*) for each experimental group/condition, given as a discrete number and unit of measurement
☐	☒	A statement on whether measurements were taken from distinct samples or whether the same sample was measured repeatedly
☐	☒	The statistical test(s) used AND whether they are one- or two-sided<br>*Only common tests should be described solely by name; describe more complex techniques in the Methods section.*
☐	☒	A description of all covariates tested
☐	☒	A description of any assumptions or corrections, such as tests of normality and adjustment for multiple comparisons
☐	☒	A full description of the statistical parameters including central tendency (e.g. means) or other basic estimates (e.g. regression coefficient) AND variation (e.g. standard deviation) or associated estimates of uncertainty (e.g. confidence intervals)
☐	☒	For null hypothesis testing, the test statistic (e.g. *F*, *t*, *r*) with confidence intervals, effect sizes, degrees of freedom and *P* value noted<br>*Give P values as exact values whenever suitable.*
☒	☐	For Bayesian analysis, information on the choice of priors and Markov chain Monte Carlo settings
☒	☐	For hierarchical and complex designs, identification of the appropriate level for tests and full reporting of outcomes
☒	☐	Estimates of effect sizes (e.g. Cohen's *d*, Pearson's *r*), indicating how they were calculated

Our web collection on statistics for biologists contains articles on many of the points above.

Software and code

Policy information about availability of computer code

Data collection	Immunostained brains were imaged using a laser-scanning confocal microscope (Zeiss LSM 780).
Data analysis	Images were processed using Fiji (version: 2.1.0/1.54j) and analyzed with custom code in python 2.7 and 3.6 and are available on github (https://github.com/ZhuoranLi97/repulsive_interactions). flyCRISPR (https://flycrispr.org/) was used for designing gRNA.

For manuscripts utilizing custom algorithms or software that are central to the research but not yet described in published literature, software must be made available to editors and reviewers. We strongly encourage code deposition in a community repository (e.g. GitHub). See the Nature Portfolio guidelines for submitting code & software for further information.

Data

Policy information about availability of data

All manuscripts must include a data availability statement. This statement should provide the following information, where applicable:

- Accession codes, unique identifiers, or web links for publicly available datasets
- A description of any restrictions on data availability
- For clinical datasets or third party data, please ensure that the statement adheres to our policy

All data are included in the manuscript and supplementary materials.

Research involving human participants, their data, or biological material

Policy information about studies with human participants or human data. See also policy information about sex, gender (identity/presentation), and sexual orientation and race, ethnicity and racism.

Reporting on sex and gender	Not applicable.
Reporting on race, ethnicity, or other socially relevant groupings	Not applicable.
Population characteristics	Not applicable.
Recruitment	Not applicable.
Ethics oversight	Not applicable.

Note that full information on the approval of the study protocol must also be provided in the manuscript.

Field-specific reporting

Please select the one below that is the best fit for your research. If you are not sure, read the appropriate sections before making your selection.

☒ Life sciences ☐ Behavioural & social sciences ☐ Ecological, evolutionary & environmental sciences

For a reference copy of the document with all sections, see nature.com/documents/nr-reporting-summary-flat.pdf

Life sciences study design

All studies must disclose on these points even when the disclosure is negative.

Sample size	No statistical tests were used to determine sample size. We used sample sizes (~4-20 flies per condition) that been previously shown to have sufficient statistical power in similar experiments in the past (e.g., Hong, Mosca, Luo 2012, Lyu, Abbott, Maimon 2022)
Data exclusions	We did not exclude flies or data from any analysis, unless brains stained for imaging appeared unsuitable (e.g., broken) at the time of imaging.
Replication	All experiments discussed in the paper were conducted on multiple animals with sample size specified. In immunostaining plots, data across multiple days were collected and all imaged brains showed the same qualitative pattern of staining. As the techinical variances are usually small, it is a standard in the field to focus on biological replicates.
Randomization	Organisms are not allocated to control and experimental groups by the experimenter in this work, rather the flies' genotype determines their group. Thus, randomization of individuals into treatments groups is not relevant.
Blinding	For counting the penetrance of the phenotype, the investigators were blind to the flies' genotypes. For the rest, the investigators were not blind to the genotype, and data collection and analysis was done computationally.

Reporting for specific materials, systems and methods

We require information from authors about some types of materials, experimental systems and methods used in many studies. Here, indicate whether each material, system or method listed is relevant to your study. If you are not sure if a list item applies to your research, read the appropriate section before selecting a response.

Materials & experimental systems

n/a	Involved in the study	
☐	☒	Antibodies
☐	☒	Eukaryotic cell lines
☒	☐	Palaeontology and archaeology
☐	☒	Animals and other organisms
☒	☐	Clinical data
☒	☐	Dual use research of concern
☒	☐	Plants

Methods

n/a	Involved in the study	
☒	☐	ChIP-seq
☒	☐	Flow cytometry
☒	☐	MRI-based neuroimaging

Antibodies

Antibodies used

rat anti-NCadherin (1:40; DN-Ex#8, Developmental Studies Hybridoma Bank), chicken anti-GFP (1:1000; GFP-1020, Aves Labs), rabbit anti-DsRed (1:500; 632496, Clontech), mouse anti-rat CD2 (1:200; OX-34, Bio-Rad), rabbit anti-HA (1:100, 3724S, Cell Signaling), mouse anti-HA (1:100, 2367S, Cell Signaling), rabbit anti-Myc (1:250, 2278S, Cell Signaling), and mouse anti-V5 (1:250, R960-25, Thermo Fisher Scientific). Secondary antibodies include: Fluorescein (FITC) AffiniPure Donkey Anti-Chicken IgY (IgG) (H+L) (Jackson ImmunoResearch 703-095-155); Donkey anti-Rabbit IgG (H+L) Highly Cross-Adsorbed Secondary Antibody, Alexa Fluor™ Plus 555 (ThermoFisher A32794); Alexa Fluor® 647 AffiniPure Donkey Anti-Mouse IgG (H+L) (Jackson Immunoresearch 715-605-151); DyLight™ 405 AffiniPure Donkey Anti-Rat IgG (H+L) (Jackson ImmunoResearch 712-475-153); Cy3-Donkey Anti-Rat IgG (H+L) (min X) (Jackson Immunoresearch 712-165-153); The anti-His-Tag Antibody coupled with iFlour 488, Genscript, A01800, used at a 1:500 dilution to detect secreted proteins from S2 cells in westerns (Ext. Data Fig. 8d,e)

Validation

All primary antibodies used in this study were validated as described at the following websites (and references therein): DSHB: https://dshb.biology.uiowa.edu/DN-Ex-8, Rockland: https://rockland-inc.com/store/Antibodies-to-GFP-and-Antibodies-to-RFP-600-901-215-O4L_23908.aspx, Takara: https://www.takarabio.com/products/antibodies-and-elisa/fluorescent-protein-antibodies/red-fluorescent-protein-antibodies?srsltid=AfmBOopUZqVextBqypoqsvRxsHH-H9rGlg0NFICn1UMie592NHF348BQ, Bio-Rad: https://www.bio-rad-antibodies.com/monoclonal/rat-cd2-antibody-ox-34-mca154.html?f=purified, Cell signaling: https://www.cellsignal.com/products/primary-antibodies/ha-tag-c29f4-rabbit-mab/3724?srsltid=AfmBOoo4fq323JT2OOUmSS57Ql81bkOHqgu_OEh2zrMiEFOwI8we-9yz; https://www.cellsignal.com/products/primary-antibodies/ha-tag-6e2-mouse-mab/2367?srsltid=AfmBOooyGtCSJAI0-x_0SG5HHyidV_FTUMprTD7bHWjkSghXfcqfT7Dw; https://www.cellsignal.com/products/primary-antibodies/myc-tag-71d10-rabbit-mab/2278?srsltid=AfmBOoqTY3_gUyCF-LzvhacFotUvpwIs3f3VWvmNzOVVtljc5Jwm6nl7; https://www.thermofisher.com/antibody/product/V5-Tag-Antibody-clone-SV5-Pk1-Monoclonal/R960-25; Validated by Genscript (https://www.genscript.com/product/documents/down?doc_name=A01800_Datasheet_Rev05.pdf&file=scm_files/productFile_notes/2025/01/07/20250107091652_A01800.pdf), and by our lab, where expression cell lines (S2 and High Five) had complete absence of signal in the media of non-transfected cells.

Eukaryotic cell lines

Policy information about cell lines and Sex and Gender in Research

Cell line source(s)

High Five cells (BTI-Tn-5B1-4) from Trichoplusia ni - for protein expression using baculoviruses - Thermo Fisher #B855-02;
Sf9 cells from Spodoptera frugiperda, used for baculovirus production (Thermo Fisher, 12659017);
S2 cells from Drosophila melanogaster (DGRC #6).

Authentication

Cell lines (from commercial source) were not authenticated, as they were only used as an exogenous production source of protein, and not studied for any biological functions.

Mycoplasma contamination

We regularly test our cell lines for mycoplasma contamination. None observed.

Commonly misidentified lines
(See ICLAC register)

None used.

Animals and other research organisms

Policy information about studies involving animals; ARRIVE guidelines recommended for reporting animal research, and Sex and Gender in Research

Laboratory animals

We used male and female Drosophila melanogaster. All fly strains and fly genotypes are described in details in the Methods. The w[1118] strain was used, with ages ranging from the larvae stage to 7 days old.

Wild animals

The study did not involve wild animals.

Reporting on sex

Experiments were performed on both sexes and reached similar conclusion.

Field-collected samples

The study did not involve samples collected from the field.

Ethics oversight

No ethical oversight was required because no vertebrates were used.

Note that full information on the approval of the study protocol must also be provided in the manuscript.

Plants

Seed stocks	Not applicable.
Novel plant genotypes	Not applicable.
Authentication	Not applicable.
